# Elevated levels of interleukin-9 in the serum of bullous pemphigoid: possible association with the pathogenicity of bullous pemphigoid

**DOI:** 10.3389/fimmu.2023.1135002

**Published:** 2023-06-15

**Authors:** Hiroshi Koga, Kwesi Teye, Arisa Sugawara, Masahiro Tsutsumi, Norito Ishii, Takekuni Nakama

**Affiliations:** ^1^ Department of Dermatology, Kurume University School of Medicine, Fukuoka, Japan; ^2^ Kurume University Institute of Cutaneous Cell Biology, Kurume University, Fukuoka, Japan

**Keywords:** autoimmune bullous disease, autoantibody, basement membrane, cytokine, pemphigoid

## Abstract

Bullous pemphigoid (BP) is an autoimmune subepidermal blistering disease (sAIBD). In addition to disease causing autoantibodies, several leukocyte subsets, including mast cells and eosinophils, play key roles in mediating skin inflammation. Detailed immunophenotyping and, more recently, the therapeutic effects of interleukin-4 (IL-4) receptor alpha inhibition in BP pointed to a prominent role of T helper 2 (Th2) cells. Among other cell types, IL-9 is expressed by Th2 and mast cells and potentially drives allergic, Th2-dominated inflammation. Although cytokines in BP have been relatively well investigated, the role of IL-9 has remained enigmatic. This study aimed to evaluate the effect of IL-9 in BP. Serum IL-9 levels were significantly elevated in patients with BP and decreased upon induction of remission. Serum IL-9 levels were not elevated in epidermolysis bullosa acquisita, another sAIBD. The time-course analysis using serum sets from four patients with BP revealed that serum IL-9 was a sensitive biomarker of BP. IL-9–positive cells infiltrated dominantly in BP lesions, especially in the blister fluid, and Th9 cells were abundant. Therefore, IL-9 was elevated in the serum and lesions of BP, which could be a biomarker of BP.

## Introduction

Bullous pemphigoid (BP), an autoimmune bullous disease, is characterized immunologically by the presence of circulating autoantibodies directed to BP180 (type XVII collagen) and BP230, which are components of hemidesmosomes at the basement membrane zone (BMZ) of the skin. Antibody binding to target proteins, especially the NC16A domain of BP180, at the BMZ is necessary for blister formation, whereas mast cell degranulation plays a key role in inflammation (1, 2). In addition to neutrophils, macrophages showed pathogenic roles *in vivo* (1). The histopathology of a lesional biopsy from patients with BP shows subepidermal splitting and a moderate-to-dense inflammatory infiltration composed of lymphocytes, neutrophils, and, most characteristically, eosinophils (1). Moreover, eosinophils can cause blistering *ex vivo* (3). Other than Immunoglobulin (Ig) G, IgE direct to BP180 and elevation of total serum IgE have been reported in BP (4). Interestingly, IgE deposition at the BMZ was associated with disease severity and treatment efficacy for BP (5). Thus, eosinophils, neutrophils, and mast cells activated by autoantibodies binding to the BMZ and cytokines that have IgG shift to IgE appear to be involved in the pathogenicity of BP. Interleukin-9 (IL-9) is expressed by T helper (Th) cell subsets, including Th2 and Th17, as well as regulatory T (Treg) cells, natural killer T cells, and mast cells. A preferential IL-9 producing Th subset was discovered and named Th9 cell (6). IL-9 exerts various effects, including the promotion of mast cell growth and activation, Th17 proliferation, transforming factor-β (TGF-β) production, and downregulation of IL-12 production in antigen-presenting cells (7, 8). Meanwhile, IL-9R knockout mice showed less suppressive effects on Treg cells and severe inflammation compared with wild-type ones in the experimental autoimmune encephalomyelitis (EAE) model (9). Some studies showed that IL-9 was involved in IgE class switch (10). IL-9 is thought to have detrimental roles in allergy such as asthma; autoimmunity, including EAE; type I diabetes; and parasitism. Recently, the anti-tumor effect of IL-9, especially to melanoma, was also reported (11). Regarding Th cell subsets and cytokines in BP, Th2, Th17, and their related cytokines, including IL-4, IL-5, IL-13, IL-17, and IL-21, have been reported (12). To the best of our knowledge, the influence of IL-9 on the pathogenesis of BP has not yet been investigated.

## Method

### Patients

Twenty-two patients with BP who were admitted to our hospital from 2013 to 2016 were enrolled in this study. Patients with BP met the following criteria: i) clinical manifestations (blistering or erythematous/urticarial plaques on the skin and/or mucosa), ii) subepidermal blistering confirmed by histopathology, iii) linear deposition of IgG and/or C3 at the BMZ as determined by direct immunofluorescence using standard procedures, and iv) positive for BP180 enzyme-linked immunosorbent assay (ELISA)/chemiluminescent enzyme immunoassay (CLEIA). Sera from patients with BP were taken at admission. Sera taken at the remission stage were also collected from the same patients. Twenty patients with epidermolysis bullosa acquisita (EBA) were enrolled in this study based on the following: i) compatible clinical features, ii) IgG reaction on the dermal side by indirect immunofluorescence of salt-split skin, and iii) detection of IgG antibodies to type VII collagen by Western blotting using a dermal extract of human skin and/or ELISA. The ethics committee of Kurume University (22008) approved the study, which was carried out in accordance with the Declaration of Helsinki guidelines.

### ELISA

Serum levels of IL-9 and total IgE were measured using the LEGEND MAX™ Human IL-9 ELISA Kit (Biolegend, San Diego, CA) and the Human IgE ELISA Quantitation Set (Bethyl Laboratories, Montgomery, TX) according to the manufacturer’s instructions, respectively. IgE antibodies directed to BP180 were measured as described previously (13). Four patients with BP who showed higher serum levels of IL-9 were selected for the time-course analysis.

### Immunohistochemistry

Formalin-fixed paraffin-embedded (FFPE) skin samples taken from skin lesions of patients with BP, atopic dermatitis, and psoriasis vulgaris and from the excision margin of skin tumors were used. FFPE sections were deparaffinized and rehydrated in xylene and 100% graded ethanol, respectively. FFPE sections were labeled with the rabbit polyclonal anti–IL-9 antibody (dilution, 1:200; ab181397, Abcam, Cambridge, MA, USA) using the Bond-III autostainer (Leica Microsystems, Newcastle, UK). Briefly, IL-9 antigen retrievals were heat-treated using epitope retrieval solution 2 (pH 9.0) for 30 min at 99°C and incubated with this antibody for 30 min at room temperature (RT). This automated system used the Refine Polymer Detection System (Leica Microsystems, Newcastle, UK) with a horseradish peroxidase polymer as a secondary antibody and 3,3′-diaminobenzidine (DAB) as the chromogen. The slides were visualized using DAB.

### Immunofluorescence

FFPE skin samples taken from the skin lesions of one patient with BP were used. FFPE skin samples taken from the lesional skin of a patient with BP were subjected to deparaffinization and dehydration by passage through xylene and an alcohol series, followed by antigen retrieval with 10 mM sodium citrate, pH 6.0 (autoclave at 120°C for 20 min). The sections were fixed with 4% paraformaldehyde for 10 min and permeabilized with 0.1% Triton X-100 for 5 min. After blocking with phosphate buffered saline (PBS) containing 1% bovine serum albumin (BSA) and 10% goat serum for 20 min, the cells were incubated with rabbit polyclonal anti–IL-9 antibody (ab181397, Abcam, Cambridge, MA) and anti-mast cell tryptase antibody (clone AA1; sc-59587, Santa Cruz Biotechnology, Dallas, TX), anti-forkhead box protein 3 (FOXP3) antibody (clone 236A/E7; Abcam, Cambridge, MA), anti-GATA binding protein 3 (GATA3) antibody (clone HG3-31; sc-268, Santa Cruz Biotechnology, Dallas, TX), anti-retinoic acid-related orphan receptor γt antibody (clone AFKJS-9; eBioscience, San Diego, CA), or anti-PU.1 antibody (clone G148-74; BD Pharmingen, San Diego, CA) for 60 min RT, followed by Alexa 488 anti-mouse IgG and Alexa 568 anti-rabbit IgG (Invitrogen, Carlsbad, CA) for 30 min RT.

### Statistical analysis

Data are presented as mean ± standard deviation (SD). Statistical calculations were performed using GraphPad Prism (version 6.05; GraphPad Software). The tests used are indicated in the figure legends. A p-value of 0.05 was considered statistically significant.

## Results

### Elevation of IL-9 levels in the serum of BP, not of EBA

**Figure 1:**
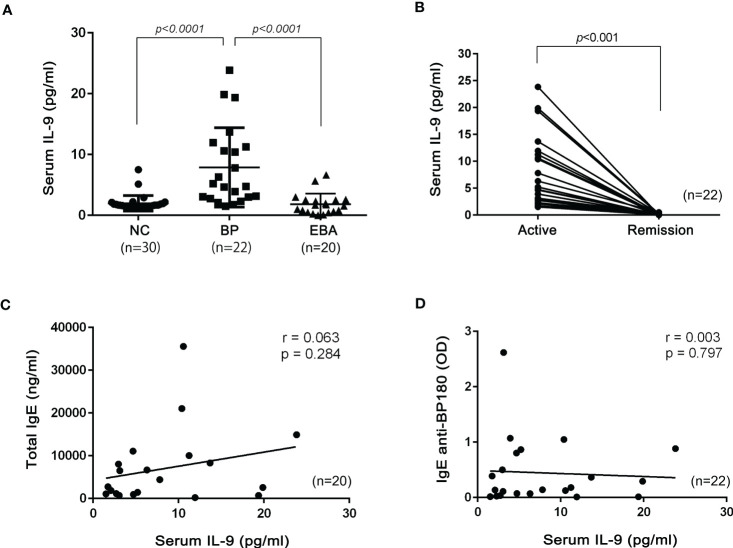
Serum IL-9 in patients with bullous pemphigoid (BP) and epidermolysis bullosa acquisita (EBA). **(A)** Serum IL-9 concentrations in normal control (NC; n = 30), BP (n = 22), and EBA (n = 20). One-way ANOVA followed by Kruskal–Wallis test was performed. **(B)** Comparison of IL-9 concentrations between patients in the active stage and in the remission stage of BP (n = 22). The Mann–Whitney test was used. The Pearson correlation test was performed with serum total IgE (**C**; n = 20) or serum IgE anti-BP180 (**D**; n = 22) and serum IL-9.

The IL-9 concentration in 22 serum samples from patients with BP and serum from healthy volunteers [normal control (NC)] was measured by commercial ELISA. Serum IL-9 levels were significantly elevated in patients with BP (**Figure 1A**). During remission, serum IL-9 levels in patients with BP decreased (**Figure 1B**). EBA is a rare and another type of AIBD whose autoantibodies target type VII collagen, a component of anchoring fibril, and share some aspects seen in BP. Notably, serum IL-9 levels in EBA were not elevated and were comparable to that in NC (**Figure 1A**).

### Serum levels of total IgE and specific IgE directed to BP180 are not related to serum IL-9 levels in BP

The relationship between serum total IgE and IL-9 levels in BP is yet to be investigated, although previous research indicated that IL-9 was involved in the IgE class switch (10). Therefore, serum levels of total IgE and IgE anti-BP180 antibodies were determined. In the analysis of the Pearson correlation test, we did not observe any significant correlations between serum total IgE and IL-9 (r = 0.063, p = 0.284) or serum IgE anti-BP180 and IL-9 (r = 0.003, p = 0.797) in BP sera (**Figures 1C, D**).

### Sensitivity of serum IL-9 as a biomarker for BP

**Figure 2:**
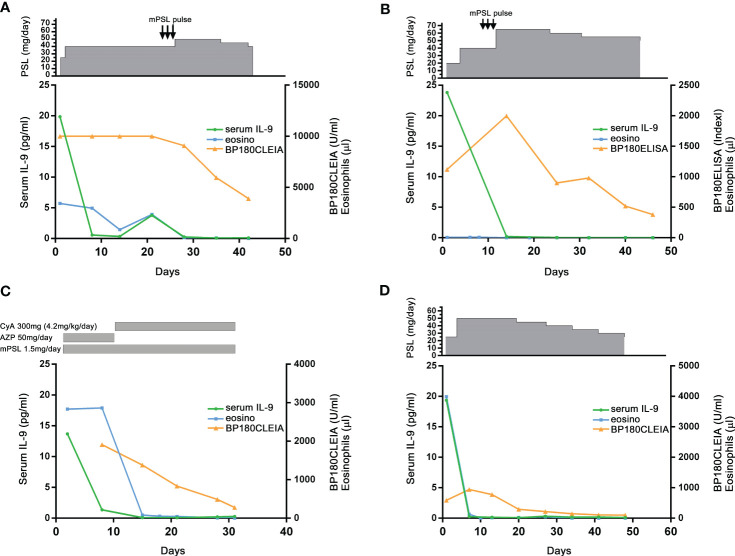
Time-course analysis of serum IL-9 levels during treatment for bullous pemphigoid (BP). In four patients with BP **(A–D)**, serum IL-9, anti-BP180 titer (ELISA or CLEIA), and the absolute count of peripheral eosinophils were measured at the initiation of the treatment every week (except case **C**). The treatments were informed on the upper section of each figure. PSL, prednisolone; mPSL, methylprednisolone; mPSL pulse, mPSL pulse therapy (1,000 mg of mPSL for 3 days); CyA, cyclosporin A; AZP, azathioprine.

The time-course analysis of serum IL-9 level was conducted using sera from four patients with BP, wherein sera were taken every week from admission. The peripheral number of eosinophils and titers for anti-BP180 IgGs was previously reported as biomarkers for BP (14). In all four cases, serum IL-9 levels rapidly decreased, which was faster than the peripheral number of eosinophils and titers for anti-BP180 IgGs (**Figures 2A–D**). Interestingly, one patient (**Figure 2A**) had relapsed clinically during the disease course and required a high intravenous dose of methylprednisolone (mPSL) pulse therapy. At the time, the serum IL-9 levels and peripheral number of eosinophils were elevated again but not the titer for anti-BP180 IgG.

### IL-9–positive cells infiltrated predominantly in BP skin lesions and blister fluid

**Figure 3:**
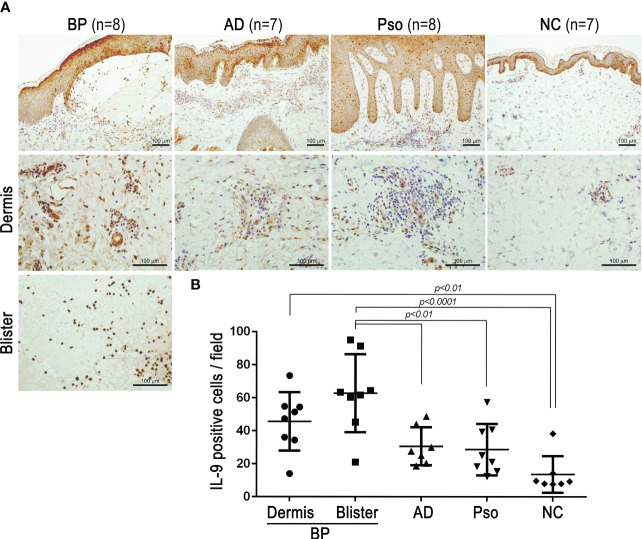
IL-9 expressions in the lesional skins of bullous pemphigoid (BP), atopic dermatitis (AD), and psoriasis vulgaris (Pso). Biopsied skin sections from BP (n = 8), AD (n = 7), Pso (n = 8), and normal control (NC; n = 7) were stained with anti-human IL-9 antibodies by immunohistochemistry. **(A)** Representative pictures with low magnification (upper panels) and high magnification in the dermis (middle panels) or blisters (lower panel) (bar = 100 µm). **(B)** IL-9–positive cells were counted at ×200 magnification. The average of counts from three fields at ×200 magnification was evaluated in each section. Data were analyzed by one-way ANOVA followed by Tukey’s test.

To visualize whether IL-9–positive cells were predominant in BP lesions, immunohistochemistry analysis with anti–IL-9 antibody was conducted. Among skin diseases, it was reported that the patient’s skin with psoriasis expressed a higher number of IL-9R–positive cells and the patient’s skin with atopic dermatitis expressed higher IL-9 mRNA compared with the normal skin (15, 16). With these findings, IL-9–positive cells infiltrated the dermis of the patients’ skin with atopic dermatitis and psoriasis, although they were insignificant. Interestingly, IL-9–positive cells infiltrated the skin, particularly in the blister fluid of patients with BP, with significance compared with NC (p < 0.0001) (**Figures 3A, B**).

### Th9 cells are the main source of IL-9 in the blister fluid of BP

**Figure 4:**
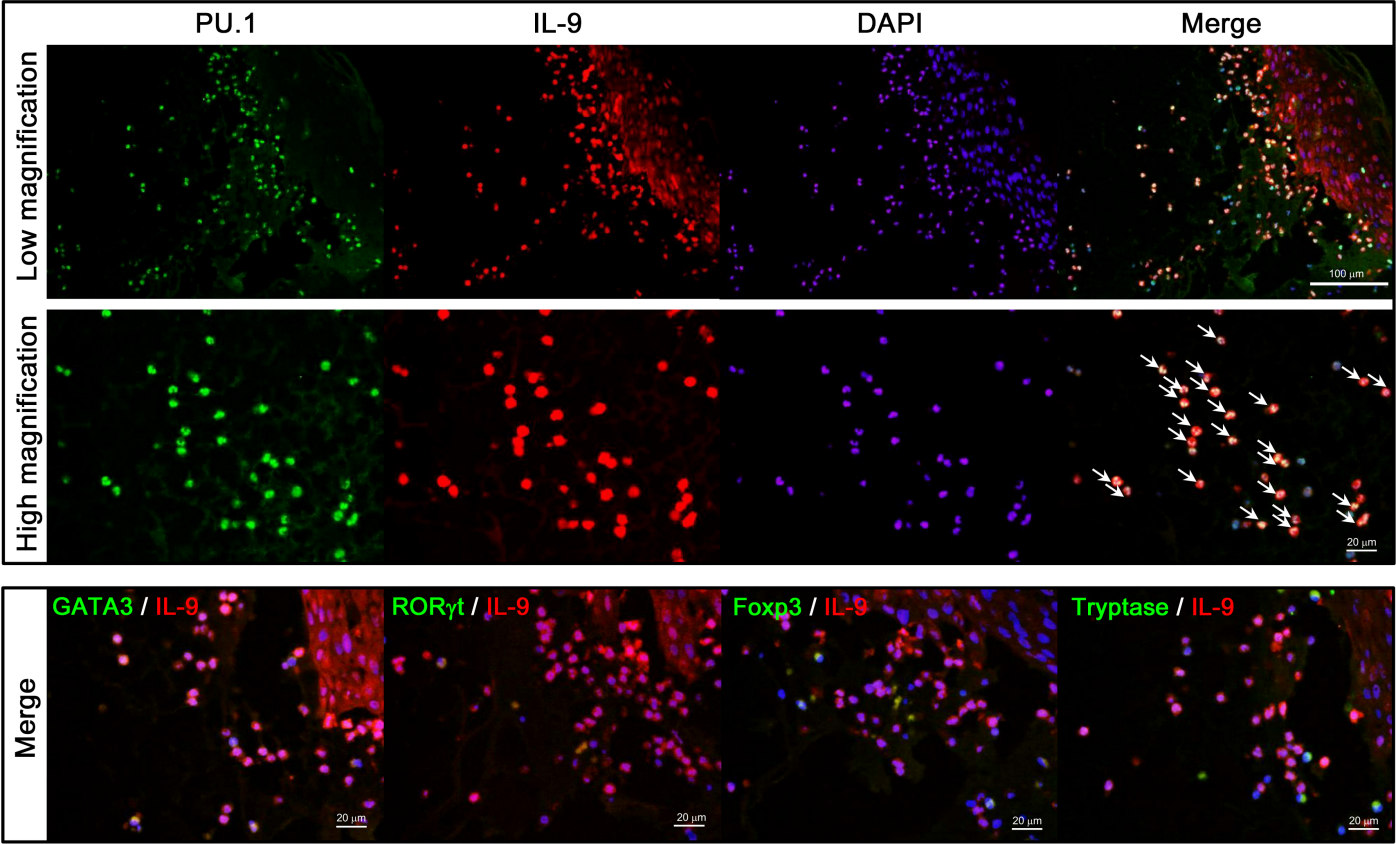
Double staining of IL-9 and transcription factors for Th2, Th17, Th9, or tryptase. A biopsied specimen from lesional BP skin was stained with combinations of anti–IL-9 (red) and the following antibodies (green): anti-PU.1 for Th9 cells (upper and middle panels), anti-GATA3 for Th2 cells, anti-RORγt for Th17 cells, anti-Foxp3 for regulatory T cells, and anti-tryptase for mast cells (lower panels), followed by 4', 6-diamidino-2-phenylindole (DAPI) to counterstain the nucleus (blue). Double-positive cells are highlighted with arrows.

IL-9 is released by Th9, Th2, Th17, Treg, and mast cells (6). To clarify which cells released IL-9 in the blister fluid of BP, lesional skin samples from patients with BP were stained with transcription factors: PU.1, GATA3, RORγt, FOXP3, tryptase, and IL-9 (**Figure 4**). IL-9–positive cells strongly expressed PU.1, the transcription factor of Th9 cell, but not GATA3, RORγt, FOXP3, and tryptase. This result revealed that Th9 cells were the main cell sources of IL-9 in the blister fluid of BP.

## Discussion

Previous studies showed the IL-9 level elevation in several dermatological diseases, including atopic dermatitis, psoriasis vulgaris, and cutaneous T-cell lymphoma (17). In atopic dermatitis, IL-9 levels correlated with clinical severity, IgE levels, and C-C motif chemokine ligand 17 (CCL17) levels (16). In psoriasis, IL-9 in induced Th17-dependent psoriasis-like skin inflammation and angiogenesis was reported (15). In mycosis fungoides, the most common form of cutaneous T-cell lymphoma, malignant and reactive T cells produce IL-9 in skin lesions and the number of producing cells decreased following a successful therapy (18). This study revealed that IL-9 levels were elevated in the serum, the skin lesion, and the blister fluid of BP and decreased in the remission stage following a successful therapy. This study revealed that Th9 cells are the main sources of IL-9 in the blister fluid of BP. Th9 cells are an important source of IL-9 and promote mast cell accumulation (8). The pathogenic role of mast cells has been reported in BP but not in EBA. The experimental EBA mice model by the injection of murine type VII collagen IgG could induce similar severity of the disease in both wild-type and mast cell–deficient mice (19, 20). Although BP and EBA share various similarities clinically and pathologically, the states of Th9 cells and releasing of IL-9, resulting in the accumulation of mast cells, appear different between these diseases. Compared with BP, cytokine balance in EBA is barely explored. The finding that blockades of IL-1/IL-1Ra, tumor necrosis factor-α (TNF-α), and IL-17R were impaired the disease in EBA mice model indicated that these cytokines were involved in the pathogenesis of EBA (21). Niebuhr et al. reported Th1 dominance in scratch-induced skin wounds in the EBA mice model (22). This study clearly indicated that IL-9 was not involved in EBA, and the distinctive trend in IL-9 level might be useful when EBA should be distinguished from BP in clinical practice.

Singh et al. reported that intradermal injection of IL-9 caused epidermal hyperplasia and dermal infiltration of CD3^+^ T cells, CD68^+^ monocytes/macrophages, and mast cells that were suppressed by pre-anti–IL-17 administration in mice. Furthermore, IL-9 injection enhanced the Th17 pathway in the psoriasis mice model (15). These results suggested that IL-9 was involved in the Th17 pathway, and Th17 cells were the main sources of IL-9 in psoriasis. Although Th17 cells and IL-17 were reported to be involved in the pathomechanism of BP (23, 24), this study showed that Th17 cells rarely produced IL-9 in the blister fluid of BP, suggesting that the pathogenic role of IL-9 differs between BP and psoriasis. Our study found no association between serum total IgE or serum anti-BP180 IgE and serum IL-9 in patients with BP, although a correlation between IL-9 and IgE levels in atopic dermatitis was reported (16). These findings suggested that IL-9 was not the primary inducer of high serum total IgE levels and the presence of IgE anti-BP180 in BP.

This study revealed that serum IL-9 level is an available biomarker to evaluate the disease activity in patients with BP. BP180 IgG and IgE levels and absolute peripheral eosinophil count are known biomarkers of BP. Among other cytokines, chemokines, and coagulation markers, eosinophil cationic protein, CXC chemokine–interleukin-8 (CXCL-8), CCL17, IL-5, IL-10, D-dimer, and F1 + 2 were also reported (14). Following treatment, serum IL-9 levels significantly decreased in patients with BP. Moreover, our fine time-course analysis showed that serum IL-9 levels rapidly decreased in all four patients after the initial treatment for treatment-naïve patients. The serum IL-9 levels and absolute peripheral eosinophil count, but not BP180 antibody, reincreased in one patient (**Figure 2A**) who experienced a flare that required additional treatment of mPSL pulse therapy. These results suggested that serum IL-9 is an incisive marker during the treatment for BP. However, further studies are needed to confirm this.

In conclusion, this study revealed that IL-9 was elevated in BP, but not in EBA, and that it is released by Th9 cells in the blister fluid. Moreover, the serum IL-9 level decreased in the remission stage of BP, suggesting its pathogenic role in BP. The distinct profile of serum IL-9 level may explain different pathomechanisms between BP and EBA. Two clinical subtypes exist in both BP and EBA, i.e., inflammatory type and non-inflammatory type (21, 25). This study could not address whether IL-9 and Th-9 cells are involved in either or both. Although IL-9 may play a role in the pathogenesis of BP, further large-scale studies are needed to confirm these findings and their clinical significance.

## Data availability statement

The original contributions presented in the study are included in the article. Further inquiries can be directed to the corresponding author.

## Ethics statement

The ethics committee of Kurume University (22008) approved the study, which was carried out in accordance with the Declaration of Helsinki guidelines. Written informed consent to use serum and histopathological samples for research purposes had previously been obtained from all patients and an opportunity for refusal to participate in this research was guaranteed by an opt-out manner.

## Author contributions

HK had full access to all the data in the study and takes responsibility for the integrity of the data and the accuracy of the data analysis. Study concept and design: HK. Acquisition of data: HK, KT, AS, and MT. Analysis and interpretation of data: HK, NI, and TN. Drafting of the manuscript: HK. Statistical analysis: HK. Obtained funding: HK. Study supervision: HK and TN. All authors contributed to the article and approved the submitted version.
